# The Efficacy and Safety of Spinal Anesthesia With Hyperbaric Ropivacaine 0.75% and Bupivacaine 0.5% in Patients Undergoing Infra-Umbilical Surgeries: A Randomized, Double-Blind Study

**DOI:** 10.7759/cureus.57005

**Published:** 2024-03-26

**Authors:** Jitendra V Kalbande, Chandini Kukanti, Habib Md R Karim, Gade Sandeep, Samarjit Dey

**Affiliations:** 1 Anaesthesiology and Critical Care, All India Institute of Medical Sciences, Raipur, Chhattisgarh, IND; 2 Neuroanaesthesiology and Critical Care, All India Institute of Medical Sciences, New Delhi, New Delhi, IND; 3 Anaesthesiology, Critical Care, and Pain Medicine, All India Institute of Medical Sciences, Deoghar, Jharkhand, IND; 4 Anaesthesiology, All India Institute of Medical Sciences, Mangalagiri, Guntur, IND

**Keywords:** 0.5% hyperbaric bupivacaine, 0.75% hyperbaric ropivacaine, cardiovascular stable, infraumbilical surgery, spinal anesthesia

## Abstract

Background: Spinal anesthesia utilizing hyperbaric 0.75% ropivacaine has been gaining clinical acceptance recently. It is a pure S-enantiomer of bupivacaine, which is expected to have a better clinical profile, but the studies for the same are yet limited. We aimed to compare the efficacy and safety of these two drugs.

Methods: Sixty patients, aged 18 to 60 years of either sex, classified as American Society of Anesthesiologists class I and II, who were undergoing elective infra-umbilical surgery, were randomly assigned to receive either 3 mL of 0.5% bupivacaine heavy or 3 mL of 0.75% ropivacaine heavy intrathecally. Efficacy parameters, including the onset and duration of sensory and motor block, time to rescue analgesia, hemodynamics, and safety in terms of complications, were recorded. We compared the data for statistical significance, considering a p-value of less than 0.05 as significant.

Results: Ropivacaine exhibited a slower onset for both sensory (153.90 ± 6.53 versus 92.46 ± 12.16 seconds; p < 0.001) and motor blockades (301 ± 6.62 versus 239.96 ± 6.27 seconds; p < 0.001). Two-segment sensory and motor blockade regression were faster with ropivacaine compared to bupivacaine (p < 0.001). However, the mean duration of sensory blockade for ropivacaine compared to that for bupivacaine (219.29 ± 15.14 versus 227.31 ± 17.20 minutes) and the requirement for rescue analgesia were not statistically different (p > 0.05). Ropivacaine also caused fewer side effects on a percentage scale.

Conclusion: In patients undergoing infra-umbilical surgery, hyperbaric ropivacaine at an equipotent dose (0.75%) proved to be a comparable and safer alternative to hyperbaric bupivacaine (0.5%). Furthermore, it had better motor-recovery profiles.

## Introduction

Spinal anaesthesia is a commonly used central neuraxial blockade often applied for lower abdominal, pelvic, and lower limb surgeries to reduce postoperative complications. Local anaesthetic agents like bupivacaine, levobupivacaine, and ropivacaine are extensively used in neuraxial nesthesia. Bupivacaine and ropivacaine both belong to the amide group of local anesthetics. However, bupivacaine is associated with higher cardiotoxicity. Ropivacaine, a pure s-enantiomer, offers the advantage of producing a more selective blockade, resulting in less motor impairment. This characteristic may facilitate early ambulation, reducing the risk of deep vein thrombosis and providing other ambulation-related benefits [1]. In terms of the minimal local anaesthetic concentration, ropivacaine demonstrates comparable potency to bupivacaine at higher dosages but exhibits lower potency at lower dosages. Consequently, when administered in a 1.5:1 ratio with bupivacaine, ropivacaine achieves a comparable level of block quality with fewer associated side effects [2].

Hyperbaric solutions are considered more predictable due to their wider and more directive distribution with lower interpatient variability [3]. Originally available only as an isobaric preparation, ropivacaine necessitates the addition of dextrose to create a hyperbaric solution when needed. However, caution is advised during the manual mixing of dextrose, as it may pose a risk of infection. Notably, ropivacaine is now commercially available as a hyperbaric solution. Nonetheless, limited evaluations are comparing the effects of 0.75% ropivacaine hyperbaric to those of equipotent hyperbaric bupivacaine, i.e., 0.5%, making it challenging to conclusively assert the advantages and disadvantages of one drug over the other.

The primary objective of the current study was to assess the efficacy of 0.75% ropivacaine hyperbaric compared to 0.5% bupivacaine hyperbaric in terms of the onset and duration of sensory and motor block. We also assessed safety by measuring the adverse effects among patients undergoing infra-umbilical surgery.

## Materials and methods

The present double-blind, randomized, active-controlled, parallel arm study was conducted following approval from the institutional ethics committee (approval number 2562/IEC-AIIMSRPR/2022, dated November 2, 2022). The study was registered with the Clinical Trial Registry of India (CTRI/2023/02/049861). Written informed consent was obtained from all patients who participated in the study, and the research was conducted according to the principles of the Declaration of Helsinki (2013) and the Good Clinical Practice guidelines.

Sixty-four adults of either sex, belonging to American Society of Anesthesiology (ASA) physical status I and II, aged 18-60 years, and scheduled for infra-umbilical surgeries using subarachnoid blocks (SABs), were enrolled. The study was conducted at an academic institute in India between April 2023 and September 2023. Patients with known allergies to any study drugs, contraindications to neuraxial block, local infection at the spinal site, bleeding diathesis, raised intracranial pressure, or for whom informed consent could not be obtained were excluded from the study. We only recruited non-pregnant and elective patients.

The pre-anesthetic check-up was conducted in the evening before the surgery. The procedure was thoroughly explained, and written consent was obtained. Patients were explained regarding the VAS (Visual Analogue Scale), used for assessing pain in the postoperative period (denoting 0 = no pain and 10 = worst imaginable pain). All patients fasted for eight hours before the scheduled operation and were premedicated with a 0.25 mg tablet of alprazolam and a 40 mg tablet of pantoprazole the night before the surgery. Randomization was achieved using a computer-generated random number table generated using www.openepi.com. The consented participants were allocated into two groups: group B, receiving 3 mL of 0.5% hyperbaric bupivacaine (15 mg), and group R, receiving 3 mL of 0.75% hyperbaric ropivacaine (22.5 mg). The principal investigator carried out the group allocation, and allocation concealment involved sealing the random numbers, which were only opened by the principal investigator. To maintain double-blinding, the principal investigator loaded the drugs, and the syringes were not labeled with the drug names but only as study drugs. The principal investigator did not participate further in the anesthesia performance or data collection. However, the statistician and one coinvestigator performed the statistical analysis and were aware of the interventions.

Upon arrival in the operating room, an intravenous line was secured on one arm with a 20G cannula, and an infusion of Ringer's lactate (RL) was initiated at a rate of 8-10 mL/kg/h of body weight. Automated monitoring of blood pressure (BP), heart rate (HR), respiratory rate (RR), pulse oximetry (SpO_2_), and electrocardiography (ECG) was performed for all patients using a multiparameter monitor of the same make. Subsequently, in the seated position and following all aseptic precautions, spinal anesthesia was performed using a midline approach at the third and fourth lumbar intrathecal spaces (L3-L4) utilizing a 26 or 27G Quincke spinal needle (B-Braun Melsungen AG, Germany) with the bevel-end facing the cephalad. The study drug was administered following the free flow of cerebrospinal fluid (CSF). Immediately after the intrathecal injection of drugs (recorded at 0 minutes), all patients were placed in a supine horizontal position.

In both groups, sensory blockade was evaluated by assessing the loss of sensation to a pinprick in the midline using a 22G blunt hypodermic needle administered every 30 seconds after the study drug. The recorded parameters included the onset of sensory block (time from injection of local anesthetic in the intrathecal space until the patient began experiencing tingling and numbness) and the total duration of sensory block (the interval from intrathecal administration until regression to the S1 dermatome). Pain and the need for rescue analgesia were assessed using the VAS score (the time from the deposition of the study drug until the requirement of first rescue analgesia when VAS > 3). Motor blockade intensity was assessed by observing the loss of antigravity movements of the legs using the Modified Bromage Scale (MBS), recorded every 30 seconds. Parameters such as onset of motor blockade (time taken in minutes from deposition of the study drug into the subarachnoid space to Bromage scale grade 1 motor block) and duration of motor block (time taken in minutes from deposition of the study drug to regression of motor block to Bromage grade 0) were noted. Modified Bromage scale (0 = no motor block, 1 = inability to raise extended leg; able to move knees and feet, 2 = inability to raise extended legs and move knees; able to move feet, 3 = complete block of motor limb).

The vital parameters such as HR, mean arterial pressure (MAP), and RR were measured every 2 minutes for 10 minutes, every 5 minutes for 30 minutes, every 15 minutes until 60 minutes, and subsequently at 60-minute intervals until 180 minutes. These data were obtained directly from the monitoring log. 

Complications, such as bradycardia, hypotension, pruritus, nausea, vomiting, shivering, local anesthetic-related toxicity, and arrhythmias, were documented. Hypotension was defined as a 20% decrease in mean blood pressure from baseline values. The baseline value for this purpose was taken as the first reading in the preoperative area. Intraoperative hypotension was addressed with fluid therapy or a single intravenous bolus dose of 6 mg of mephentermine. Bradycardia for this study was considered at a heart rate of less than 50 beats per minute and was treated with an intravenous atropine dose of 0.6 mg. During the postoperative period, hemodynamic monitoring continued. Any heart rhythm disturbance other than sinus bradycardia and tachycardia was noted as arrhythmia. Further, nausea, vomiting, and shivering were. also noted. Patients were monitored for regression from the sensory and motor blockade based on the Modified Bromage scale, which was assessed every 10 minutes, and the requirement of the first rescue analgesic (intravenous paracetamol 1 g was administered) as guided by the VAS scale, which was assessed at the same time or on patients requests for analgesics/complaints of pain.

The sample size was calculated based on the standard deviation (SD) and minimum difference detected for the mean time to complete motor blockade in the study by Gaikwad et al. [4], where the minimum difference between the two groups was equal to 0.5 (SD = 1). With a 95% confidence interval and 80% power, the total sample size was 32 for each group, 64 in total. The formula used for sample size calculation was (*N* = 2*(*Zα* + *Zβ*)2 * *σ*2 / *δ*2), where *N* is the sample size for each group, *Zα* equals 1.96 at the 95% confidence interval, and *Zβ* equals 0.84 at 80% power.

The master chart was prepared in Microsoft Excel (Microsoft® Corp., Redmond, WA), and the statistical analysis was carried out using Statistical Packages for the Social Sciences (SPSS) version 22 for Windows (IBM SPSS Corp., Armonk, New York, USA). Continuous and categorical variables are expressed as the mean ± SD and percentages. Descriptive statistics were done to determine the distribution of age, gender, and ASA. An independent t-test was used to compare age, height, weight, duration of surgery, hemodynamic parameters, block parameters such as onset, duration, and recovery time of the sensory block, time to two-segment regression, and rescue analgesia. The chi-square test was performed to compare the highest-level block and complications. Two-sided p-values were considered statistically significant at p < 0.05.

## Results

**Figure 1:**
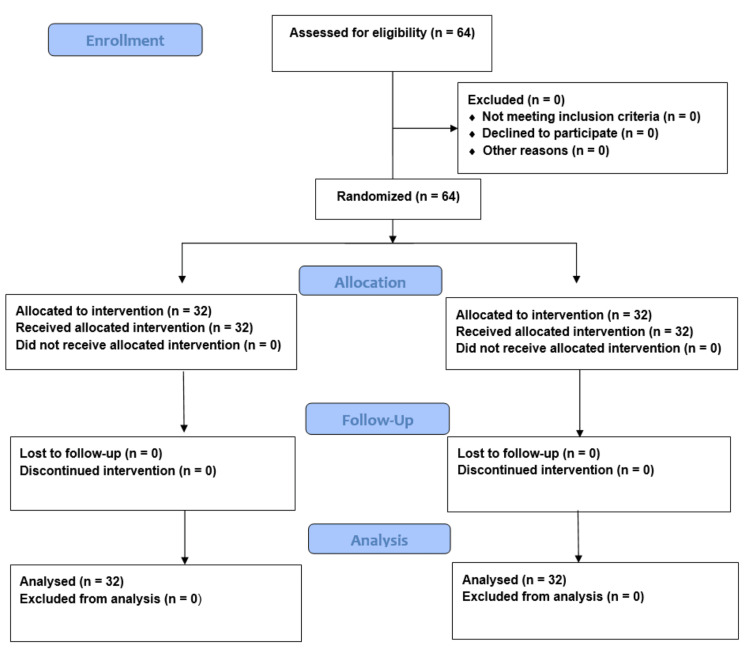
Consort flow diagram.

A total of 60 patients were analyzed. A consort flow diagram is shown in Figure 1.

The physical characteristics of the two groups in terms of age, sex, ASA status, height, weight, and duration of surgery were comparable (Table 1).

**Table 1:** Physical characteristics and duration of surgery in both groups. Values are presented as mean ± standard deviation. *P > 0.05 not significant. M: male, F: female, ASA: American Society of Anesthesiologists, SD: standard deviation.

Parameter	Group B (N=32)	Group R (N=32)	P-value
Age (years), mean (SD)	39.46 ± 11.29	41.50 ± 9.84	0.446*
Gender (M/F)	20/12	17/15	0.448*
ASA physical status (I/II)	14/18	15/17	0.802*
Height (cm), mean (SD)	164.12 ± 2.56	165.09 ± 2.96	0.167*
Weight (kg), mean (SD)	62.68 ± 4.42	63.65 ± 4.24	0.375*
Duration of surgery (minutes)	87.50 ± 4.77	89.25 ± 4.97	0.156*

According to our study, the mean onset time of sensory blockade was significantly faster in group B (92.46 ± 12.16 seconds) than in group R (153.90 ± 6.53 seconds) (p < 0.001). Similarly, the mean onset time of motor blockade was shorter in group B (239.96 ± 6.27 seconds) than in group R (301 ± 6.62 seconds) (p < 0.001). The time to reach the maximum extent of cephalad spread and the achieved level were similar in both groups. Additionally, two-segment sensory regression occurred faster in the ropivacaine group (91.12 ± 5.08 min) than in the bupivacaine group (100.65 ± 6.87 minutes; p < 0.001) (Tables 2-3).

**Table 2:** Comparison of highest-level block. Chi-square test; *P > 0.05 not significant.

Highest level block	0.5% Bupivacaine		0.75% Ropivacaine		P-value
	N	%	N	%	
T6	3	9.4	2	6.3	0.884*
T8	19	59.4	19	59.4	
T10	10	31.3	11	34.4	

**Table 3:** Comparison of sensory and motor blockade between the two groups. Values are presented as mean ± standard deviation. Independent t-test; **P < 0.001 highly significant, *P > 0.05 not significant.

Parameters	0.5% Bupivacaine		0.75% Ropivacaine		P-value
	Mean	SD	Mean	SD	
Onset of sensory block (seconds)	92.46	12.16	153.90	6.53	<0.001**
Onset of motor block (seconds)	239.96	6.27	301.00	6.62	<0.001**
Time taken to achieve T10 sensory block (minutes)	5.12	0.70	6.73	0.66	<0.001**
Time taken to achieve complete motor block (minutes)	9.55	0.51	12.50	0.65	<0.001**
Time to two-segment regression (minutes)	100.65	6.87	91.12	5.08	<0.001**
Complete sensory recovery (S1) (minutes)	227.31	17.20	219.29	15.14	>0.052*
Complete motor recovery (minutes)	196.75	17.75	159.06	11.61	<0.001**
Rescue analgesia (minutes)	241.14	8.75	237.63	6.19	>0.068*

**Figure 2:**
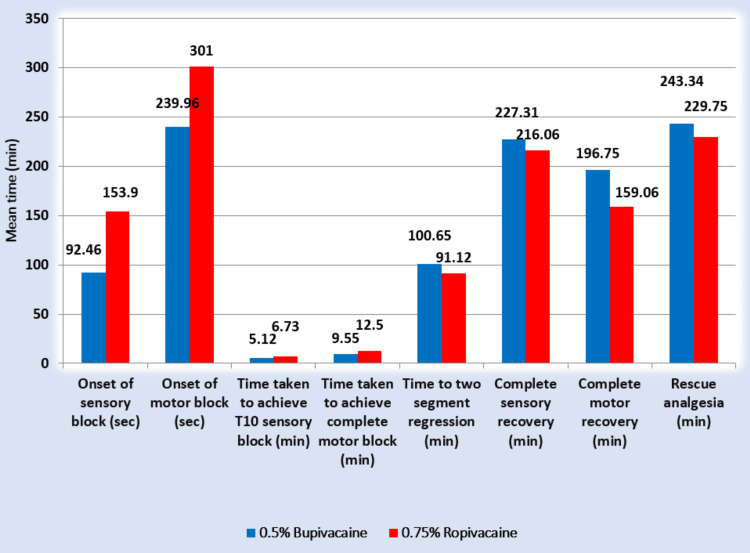
Bar graph, showing a mean comparison of efficacy endpoints between 0.5% bupivacaine and 0.75% ropivacaine.

The time required for complete recovery of motor block in the bupivacaine group was longer (196.75 ± 17.75 minutes) compared to ropivacaine (159.06 ± 11.61 minutes; p < 0.001). The mean durations of sensory blockade (S1) in group B (227.31 ± 17.20 minutes) and group R (219.29 ± 15.14 minutes) were comparable but not statistically significant (p > 0.05). The requirements for rescue analgesia in both groups were also longer and statistically non-significant (p > 0.05) (Figure 2).

**Figure 3:**
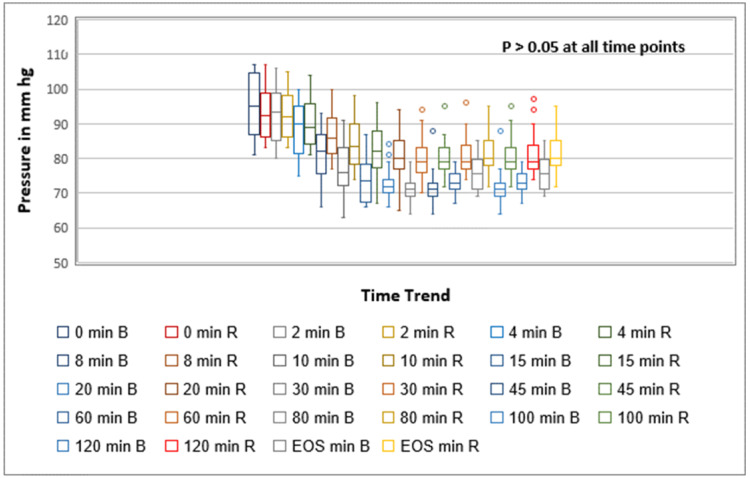
Box and whisker plot showing trend of mean blood pressure among the groups. The timepoints are in minutes. Box and whisker show maximum, minimum, mean, and interquartile ranges. EOS: end of surgery; B: 0.5% bupivacaine; R: 0.75% ropivacaine.

**Figure 4:**
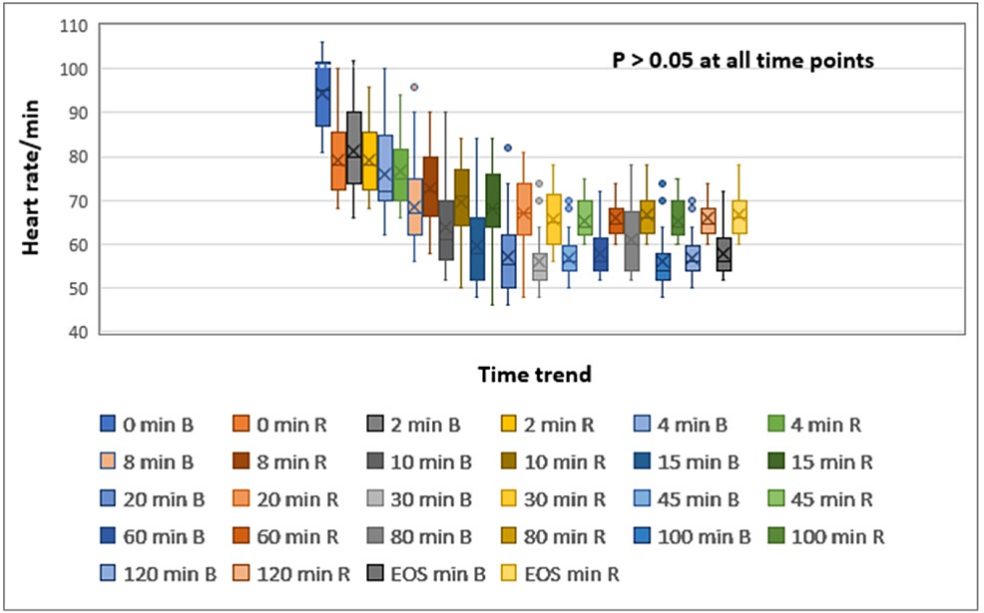
Box and whisker plot showing trend of heart rate among the groups. The timepoints are in minutes. Box and whisker show maximum, minimum, mean and interquartile ranges. EOS: end of surgery; B: 0.5% bupivacaine group; R: 0.75% ropivacaine group.

Hemodynamic changes were not significant when compared between the two groups (p > 0.05). However, the mean arterial pressure and heart rate were consistently higher throughout all time intervals in the ropivacaine group than in the bupivacaine group (Figures 3-4). 

The incidence of bradycardia, nausea, vomiting, and shivering during the intraoperative period did not differ significantly between the two groups (Table 4).

**Table 4:** Complications. Chi-square test; *p > 0.05 not significant.

Complications	0.5% Bupivacaine		0.75% Ropivacaine		P-value
	N=32	%	N=32	%	
Hypotension	7	21.87	2	6.25	0.884*
Bradycardia	4	12.5	1	3.12	
Shivering	3	9.37	1	3.12	
Nausea and vomiting	3	9.37	0	0	

## Discussion

One of the primary goals of perioperative physicians while performing SAB is to achieve adequate effects for the required duration without causing significant derangement of physiological parameters or side effects. Our study employing equipotent ropivacaine and bupivacaine for SAB in patients undergoing infra-umbilical surgeries revealed that ropivacaine 0.75% heavy can achieve the same effect when used instead of bupivacaine 0.5% heavy. However, the onset was slower for ropivacaine by a few minutes. However, the minimal prolonged duration is unlikely to affect healthcare dynamics and is practically acceptable in most elective surgeries. Nonetheless, this approach might be a hurdle for time-sensitive cases such as emergency cesarean sections.

Till now, hyperbaric bupivacaine (specific gravity, 1.025) has been the primary choice for SAB. It is favored for offering profound sensory analgesia, adequate muscle relaxation, reduced operative blood loss, and postoperative pain relief. However, caution is warranted due to the potential cardiotoxicity and neurotoxicity of the R-enantiomer [2]. Therefore, there is a need for a local anesthetic with minimal side effects while ensuring sufficient sensory and motor blockade [3]. Ropivacaine, a long-acting amide local anesthetic agent, is a pure S (−) enantiomer of bupivacaine. It exhibits reduced potential for cardiotoxicity and neurotoxicity, rendering it a safer alternative to the racemic preparation, bupivacaine [5]. In scenarios like epidural analgesia or spinal anesthesia, where low doses are employed, ropivacaine exhibits lower potency than bupivacaine. However, at higher doses, particularly in peripheral nerve blocks, the potency and efficacy of both agents seem comparable [5]. A comparison of identical doses of isobaric ropivacaine and bupivacaine revealed that ropivacaine exhibits nearly equivalent efficacy but with variable or inadequate block characteristics and a shorter duration of sensory and motor block [6].

When administered in a 1:1.5 dose ratio, bupivacaine and ropivacaine yield almost comparable block characteristics to the two local anesthetics [1]. It is known that hyperbaric local anesthetics offer a more predictable spread and consistent sensory and motor block. Adding glucose increases the density, facilitating a more even distribution of local anesthetics. This effect is attributed to gravity's influence on the drug bolus's spread along the lumbar curve slope in the supine position [7]. While earlier studies created hyperbaric ropivacaine by adding dextrose to isobaric ropivacaine, our study utilized commercially available hyperbaric ropivacaine (0.75%) (specific gravity, 1.025-1.035) (by NEON Laboratories, Mumbai, India) [4].

This comparative study examined the effectiveness (0.75%) of hyperbaric ropivacaine compared to hyperbaric bupivacaine (0.5%) among patients undergoing infra-umbilical surgeries under spinal anesthesia. It revealed that equipotent doses of 0.75% hyperbaric ropivacaine (3 mL) and hyperbaric bupivacaine (3 mL) demonstrated statistically comparable efficacy, with the former exhibiting a similar safety profile.

In the present study, no differences were observed between the two groups in age, sex, weight, height, ASA physical status, or duration of surgery. However, the characteristics of the nerve blocks, including the onset of sensory and motor blockade, time taken to achieve T10 sensory block, and complete motor block, were delayed in the ropivacaine group compared to those in the bupivacaine group. Our findings align with those of Gaikwad et al. [4] and Mahajan and Patel [8], who reported delayed sensory and motor block onset in the ropivacaine group. This delay may be attributed to the less lipophilic nature of ropivacaine. Given that the effectiveness of local anesthetics is directly related to the myelination and size of nerve fibers, more lipophilic local anesthetics (such as bupivacaine) are likely to penetrate larger myelinated motor fibers more rapidly and persistently than less lipophilic drugs (such as ropivacaine).

In the current study, we found that the level of sensory block achieved was comparable in both groups. Nevertheless, there was a faster regression of the two segments in the ropivacaine group, consistent with findings in other studies [4,8]. An interesting finding in our study was that, despite the quicker regression of two segments in the ropivacaine group, the total duration of sensory block and the time required for rescue analgesia were comparable in both groups. The ropivacaine group maintained a good sensory block until the conclusion of surgery. Similarly, Kallio et al. reported that ropivacaine 15 mg provided a duration of sensory block similar to bupivacaine 10 mg [9]. This equivalence is attributed to the fact that at equipotent doses (1.5:1 or 0.75%), ropivacaine exhibits comparable potency to bupivacaine at higher dosages [10]. Additionally, it is well established that hyperbaric solutions, as opposed to plain local anesthetic solutions, result in a more predictable cephalad spread and prolong the duration of clinically useful blocks.

Our observations also revealed that motor blockade was prolonged in the bupivacaine group. Being less lipophilic, ropivacaine exerts a greater effect on non-myelinated pain fibers than myelinated motor fibers, leading to a pronounced degree of sensory-motor differentiation compared to bupivacaine [11]. This differentiation contributes to early patient movement, a phenomenon supported by similar observations in other studies [5,8,9,11].

The observed common side effects in both groups included hypotension and bradycardia. The bupivacaine group showed a numerically higher incidence of hypotension and bradycardia, although the difference between the two groups was not statistically significant. Several authors, such as Dar et al., Whiteside and Wildsmith, and Gadre et al., have corroborated this finding in their studies, indicating a lower incidence of hypotension with intrathecal ropivacaine compared to intrathecal bupivacaine [12-14]. This difference is attributed to the absence of the R enantiomer in ropivacaine [15,16]. As ropivacaine is a pure S-enantiomer, it results in less hemodynamic instability during surgery. The remaining intraoperative complications (nausea, vomiting, and shivering) showed no statistically significant differences between the two groups.

Considering the minimal impact of ropivacaine on hemodynamics with fewer adverse effects, it can be considered an alternative to bupivacaine for spinal anesthesia in patients with heart disease and elderly patients with multiple comorbidities. Early recovery of motor function is associated with a decreased incidence of venous thromboembolism and facilitates early mobilization.

Our study has the strength of randomization and an adequate sample size. However, it also has notable limitations: it focuses on relatively healthy adults, and the effects on older patients with cardiovascular comorbidities remain unknown. The study did not standardize the dose based on age, height, and weight. Furthermore, we performed a study on elective surgeries only, and the practical application of the results needs to be weighed against time-sensitive emergency cases. A single-center study is also another limitation.

## Conclusions

In conclusion, 0.75% hyperbaric ropivacaine, administered intrathecally at a dosage of 22.5 mg, has been demonstrated to offer clinically effective anesthesia for elective infra-umbilical surgeries. At an equipotent dose, it exhibits a comparable duration of sensory blockade, consistent blockade height, stable hemodynamics, a shorter duration of motor blockade, and adequate postoperative analgesia compared to hyperbaric bupivacaine. This facilitates early ambulation, enhances patient satisfaction, and suggests its potential as an efficient and equally safe alternative to the more commonly used bupivacaine in routine clinical practice.
